# Comparing time series transcriptome data between plants using a network module finding algorithm

**DOI:** 10.1186/s13007-019-0440-x

**Published:** 2019-06-01

**Authors:** Jiyoung Lee, Lenwood S. Heath, Ruth Grene, Song Li

**Affiliations:** 1 Genetics, Bioinformatics and Computational Biology, Virginia Polytechnic Institute and State University, Blacksburg, VA 24061 USA; 2 Department of Computer Science, Virginia Polytechnic Institute and State University, Blacksburg, VA 24061 USA; 3 School of Plant and Environmental Sciences, Virginia Polytechnic Institute and State University, Blacksburg, VA 24061 USA

**Keywords:** Comparative transcriptome analysis, Network, Sequence homology, Arabidopsis, Soybean, Embryo development

## Abstract

**Background:**

Comparative transcriptome analysis is the comparison of expression patterns between homologous genes in different species. Since most molecular mechanistic studies in plants have been performed in model species, including Arabidopsis and rice, comparative transcriptome analysis is particularly important for functional annotation of genes in diverse plant species. Many biological processes, such as embryo development, are highly conserved between different plant species. The challenge is to establish one-to-one mapping of the developmental stages between two species.

**Results:**

In this manuscript, we solve this problem by converting the gene expression patterns into co-expression networks and then apply network module finding algorithms to the cross-species co-expression network. We describe how such analyses are carried out using bash scripts for preliminary data processing followed by using the R programming language for module finding with a simulated annealing method. We also provide instructions on how to visualize the resulting co-expression networks across species.

**Conclusions:**

We provide a comprehensive pipeline from installing software and downloading raw transcriptome data to predicting homologous genes and finding orthologous co-expression networks. From the example provided, we demonstrate the application of our method to reveal functional conservation and divergence of genes in two plant species.

## Background

Expression analysis is commonly used to understand the tissue or stress specificity of genes in large gene families [1–5]. The goal of comparative transcriptome analysis is to identify conserved co-expressed genes in two or more species [3, 6, 7]. The traditional definition of orthologous genes is based solely on sequence homology [8–11] and syntenic relationships [2, 12–14] and not at all on gene expression patterns. In contrast, comparative transcriptome analysis combines a comparison of gene sequences with a comparison of expression patterns between homologous genes in different species. Homologous genes have been reported to be expressed at different developmental stages, in different tissue types, or under different stress conditions [3, 15–17]. This documented divergence of expression patterns provides crucial evidence for the existence of functional divergence of homologous genes across species [18, 19]. Therefore, comparative transcriptome analysis is an important tool for distinguishing those genes that have retained functional conservation from those that have undergone functional divergence. Comparative transcriptome analysis is particularly important for plant research, since most molecular mechanistic studies in plants have been performed in model species, primarily *Arabidopsis thaliana* [20]. The consequence of this narrow focus is that the functional annotation of the genes of many other plant species relies solely on sequence comparisons with Arabidopsis [21].

To compare transcriptomes between any two species, a first step is to establish homologous relationships between proteins in the two species. A second step is to identify expression data obtained from experiments that are performed under similar conditions or tissue types. The third step is to compare the expression patterns between the two data sets. In this protocol, we will compare published time course seed embryo expression data from Arabidopsis [22] with data from the same tissue in soybean [23] as a demonstration of how to apply computational tools to comparative transcriptome analysis.

In contrast with the time course data examined here, many other data sets have been reported from “treatment–control” experiments (one time point only and two treatment conditions). For example, soybean roots were treated with drought stress in one experiment [4]. To address the question of functional conservation versus functional divergence within gene families, these soybean root data can be compared with transcriptome data from Arabidopsis roots, under a similar stress [24]. This is a relatively simple problem, because, in both experiments, we can identify lists of differentially expressed genes in response to the same or similar treatments. It is a simple two-step process to identify conserved co-expressed genes for treatment–control experiments. First, one needs to identify a list of gene pairs that are homologous between these two species. A simple BLAST search or other more sophisticated approaches, such as OMA, EggNog, or Plaza [9, 10, 12], can be used to identify homologous genes. Second, the two lists of differentially expressed genes can be compared to find whether any pairs of these homologous genes appear in both lists.

In this article, we are focusing on a more complex scenario: two time-series experiments were performed for the same developmental process in two different species [25]. Time course data provide more data points than simple treatment–control experiments and, thus, can reveal relationships based on development between homologous genes in two organisms. However, this is also challenging, because the number of time points in the two experiments are different. It can be challenging to precisely match developmental stages between two species, although some excellent approaches have been proposed [25, 26]. Despite the difficulty of establishing a one-to-one mapping between the developmental stages of two species, many biological processes, such as embryo development, are known to be highly conserved between different plant species that are compared in comparative transcriptome analysis [27, 28]. One way to solve this developmental stage problem is to convert the gene expression patterns into a co-expression network and then apply network alignment or network module finding algorithms to these co-expression networks [29]. Transforming expression data to a network form simplifies the problem and allows exploration using well established network algorithms [30, 31]. Here, we describe how to perform such analysis using a published simulated annealing method [29]. We also discuss how to visualize the resulting co-expression networks across species [32] and the results from different choices of homology finding methods.

## Results

### Comparative transcriptome analysis overview

**Fig. 1:**
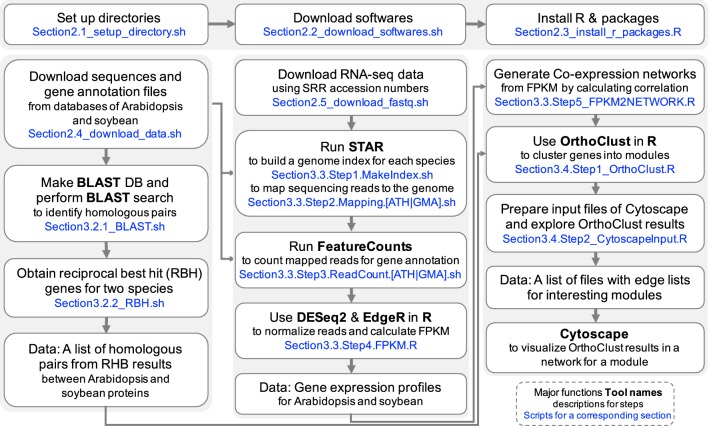
A workflow of comparative transcriptome analysis between soybean and Arabidopsis. It is composed of three major parts: identification of ortholous pairs between two species using BLAST, RNA-seq analysis to get co-expression networks, and running OrthoClust to cluster genes with orthologous relations. Blue fonts indicates softwares or scripts used in this workflow

**Fig. 2:**
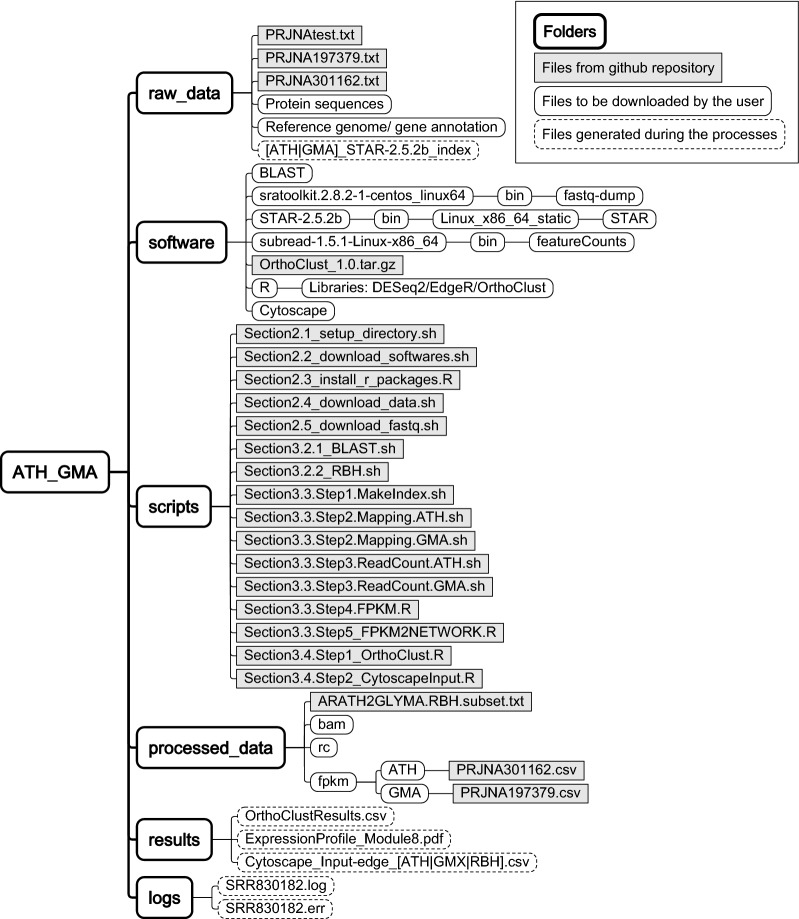
Folder structure for data analysis

This protocol provides details of comparative transcriptome analysis between two species. We not only compute sequence similarity between protein coding genes in two species, we also integrate the gene expression patterns of these genes from two different species under similar biological processes. There are three major steps in this analysis (Fig. 1): (1) identify homologous genes between two species; (2) generate a gene expression data matrix and a co-expression network in each species; (3) perform cross species comparisons of gene homology and expression patterns. For each of these steps, multiple bioinformatics tools are available. This protocol will provide a basic workflow for each of the steps and the reader can substitute individual steps with other tools (see “Discussion” section). To facilitate reproducible and effective computational analysis [33, 34], we suggest that the user creates a folder structure (Fig. 2) such that the raw data, processed data, results, and scripts for data processing can be organized into their respective folders.

#### Obtaining reciprocal best hit (RBH) genes

Reciprocal best BLAST hit (RBH) is a commonly used method to identify homologous genes in two species [35–41]. To identify RBH genes between any two species, the BLAST results from protein sequence alignment were first parsed to identify the best BLAST hit for each soybean protein in the Arabidopsis protein list. For each soybean protein, there was at most one best BLAST hit protein in the Arabidopsis proteome. For each of the Arabidopsis proteins identified in the first step, the best BLAST hit of each protein in the soybean proteome was also identified. If this best hit was also the original homologous gene found in the first step, this pair of proteins was defined to constitute an RBH pair.

For genes with multiple isoforms and potentially multiple protein sequences, we performed the BLAST analysis at the isoform level and then collapsed all the isoforms for each gene to find the best match. We developed a Python script that can identify RBH genes from the above two species from BLAST results. The user can download this script from a GitHub repository for this pipeline (https://github.com/LiLabAtVT/CompareTranscriptome.git). Although RBH genes are widely used in comparative genomic analysis, other methods can be used to identify homologous genes for downstream analysis (see “Discussion” section). An example file (ARATH2GLYMA.RBH.subset.txt) of RBH genes is provided. The user can use this file to perform the following analysis without running the RBH script. The summary statistics for the RBH analysis results are provided in Table 1. We found 13,024 RBH pairs in these two species and these genes pairs were used in this analysis.

**Table 1:** Results of Identified Orthologous Genes

Species	Soybean	Arabidopsis
Number of proteins (Total number of gene models)	48,375 (56,044)	24,148 (37,336)
Blast results in each species (Query: Blast DB)	1,086,080 (Soybean: Arabidopsis)	1,081,623 (Arabidopsis: Soybean)
Number of RBH genes in each species	13,024	13,024
Number of 5 best hit in each species	208,343	112,819

### Co-expression networks

Co-expression network generation was followed by gene expression data processing steps using the same pipeline for both species. For this step, we used 1267 Arabidopsis genes and 2092 soybean genes that are known to be essential for embryo development in Arabidopsis [27, 42] and soybean [23]. To convert the gene expression profiles into gene co-expression networks, we first filtered genes with low expression levels and low variation across conditions from the gene expression profiles. After that, we calculated gene co-expression matrices using the Pearson Correlation Coefficient (PCC). PCC and the p-values of PCC were used to filter genes (see “Methods” section). From a total of 24,148 Arabidopsis genes, 1267 genes were selected for the co-expression network analysis. After filtering by PCC and p-values, 1092 genes remained and were used to construct a co-expression matrix. A total of 595,686 co-expression edges were initially generated from the PCC step; 17,648 co-expression edges among 853 genes remained after filtering. For the soybean co-expression network, 62,185 co-expression edges among 1401 genes were finally obtained.

### OrthoClust analysis

From the previous steps, we generated two co-expression networks and a list of homologous pairs for the selected genes of these two species as inputs to the OrthoClust analysis. The examples of three input data files are provided in Table 2. OrthoClust integrated gene co-expression networks of Arabidopsis and soybean with orthologous pairs of the genes from two species and clustered three kinds of relations into cross-species modules. From one trial of the OrthoClust analysis, we obtained 353 modules and ranked them according to the total number of genes from a module as an example (Table 3). Some modules contain genes from both species with some level of balance, and other modules have one species only.

**Table 2:** Examples of input data files for OrthoClust analysis

(A)		(B)		(C)	
Row	Column	Row	Column	Soybean gene	Arabidopsis gene
Glyma.01G006400	Glyma.01G016500	AT1G01540	AT1G05350	Glyma.01G001300	AT2G07050
Glyma.01G021300	Glyma.01G021400	AT1G06040	AT1G06150	Glyma.01G005800	AT4G29310
Glyma.01G019400	Glyma.01G022500	AT1G01720	AT1G07400	Glyma.01G006100	AT4G26300
Glyma.01G015400	Glyma.01G026700	AT1G05230	AT1G07570	Glyma.01G010100	AT1G32090
Glyma.01G025100	Glyma.01G026700	AT1G02660	AT1G08230	Glyma.01G015400	AT2G35470
Glyma.01G025100	Glyma.01G028900	AT1G01090	AT1G08510	Glyma.01G019400	AT5G65670

There are three inputs: two co-expression networks of (A) soybean and (B) Arabidopsis, (C) orthologous pairs between soybean and Arabidopsis.

**Table 3:** Top 10 OrthoClust results sorted by the total number of genes from a module

No.	Module ID	Total number of genes from a module	The number of genes from soybean	The number of genes from Arabidopsis
1	2	352	273 (77.6%)	79 (22.4%)
2	8	331	255 (77.0%)	76 (23.0%)
3	39	297	174 (58.6%)	123 (41.4%)
4	1	253	214 (84.6%)	39 (15.4%)
5	3	245	207 (84.5%)	38 (15.5%)
6	187	215	56 (26.0%)	159 (74.0%)
7	224	212	53 (25.0%)	159 (75.0%)
8	57	192	110 (57.3%)	82 (42.7%)
9	113	147	38 (25.9%)	109 (74.1%)
10	19	45	39 (86.7%)	6 (13.3%)

OrthoClust was performed with parameters κ = 3, gene co-expression correlation cutoff ≥ 0.99 and homologous pairs obtained from RBH Blast

#### Visualization of OrthoClust results as a network

To visualize OrthoClust results, we used Cytoscape, a network visualization platform to analyze biological networks and to integrate multiple data into networks such as gene expression profiles or annotation [43]. We used module 8 from the previous step as an example. There are three input files: (1) the soybean co-expression network edge list for genes in module 8, (2) the Arabidopsis co-expression network edge list for genes in module 8, and (3) the RBH list for genes in module 8.

**Fig. 3:**
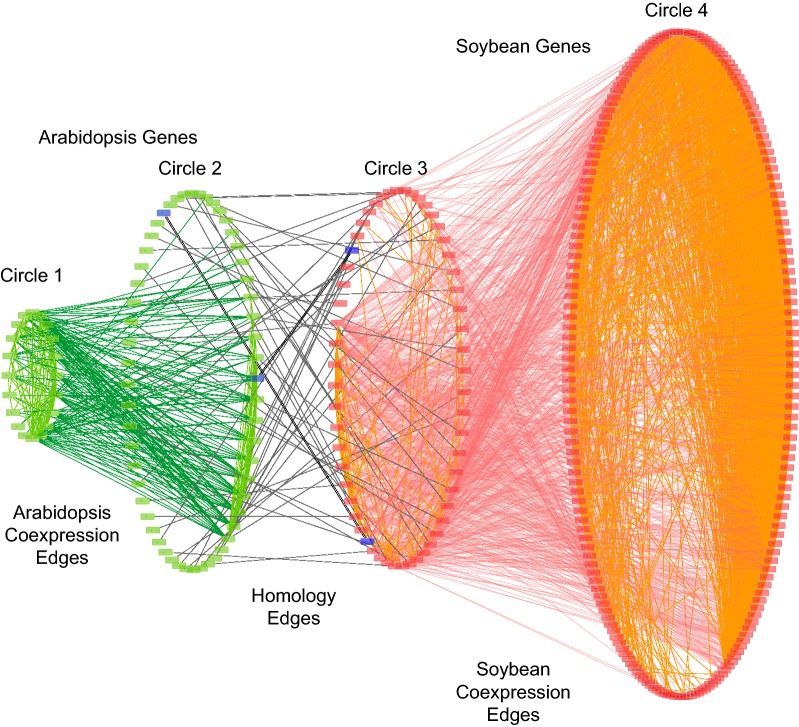
Visualization of module 8 from OrthoClust result. In this network, Circle 1 and 4 stand for groups of genes from Arabidopsis and soybeans that do not have orthology in the other species and only co-expression partner from the same species. Circle 2 and 3 denote genes have orthologous partner in the other species as well as their co-expression partners from the same species. Green nodes are genes from Arabidopsis, and red from soybean. Edges from co-expression network of Arabidopsis are green, and those of soybeans are red. Black double lined edges indicate homologous pairs between soybean and Arabidopsis genes. Four genes from raffinose biosynthesis pathways are highlighted in blue color and their homologous pairs have thicker edges

As an example of the network with module 8, nodes and edges from soybean and Arabidopsis genes were indicated by green and orange colors respectively. To highlight genes of interest, we used thicker double lines for edges and blue color for nodes. We separated genes into four groups according to their input files and species and laid out each of them with a Degree Sorted Circle Layout (Fig. 3).

#### Visualization of OrthoClust results as expression profiles

**Fig. 4:**
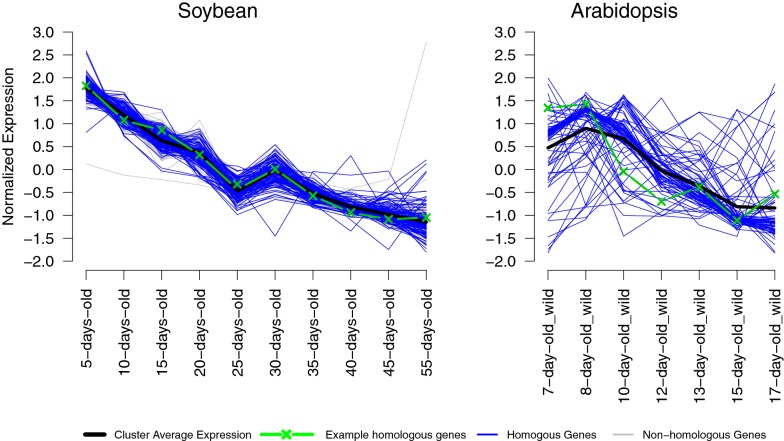
Expression plots of genes from Arabidopsis and soybean bellowing to one of modules of OrthoClust result. One example of homologous genes in Arabidopsis and soybeans are AT5G52560 and Glyma.04G245100 are highlighted in green

To understand expression patterns of genes from the selected modules under different stages of embryo development, we visualized gene expression profiles for module 8 (Fig. 4). In this module, most soybean genes are tightly clustered. Some Arabidopsis genes are tightly clustered (close to the black line) whereas other Arabidopsis genes are not. This result shows that many genes in the soybean co-expression cluster change their expression patterns in Arabidopsis, suggesting potential functional divergence of these genes. In contrast, many genes that are RBH pairs in the two species have similar expression patterns. For example, one gene (AT5G52560, green line) that is related to the raffinose biosynthetic pathway has a similar decreasing expression pattern as its RBH gene (Glyma.04G245100) in soybean.

### Effect of different parameters in OrthoClust analysis

**Fig. 5:**
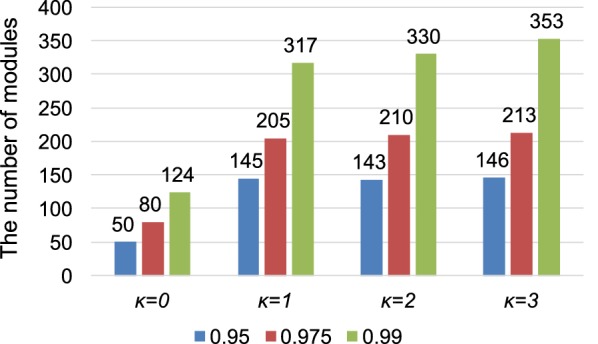
Effect of different correlation cutoff and $\kappa$ values on the number of modules in orthoClust analysis

We analyzed how different parameters affect the results of this analysis. We focus on two parameters (Fig. 5), the Pearson Correlation Coefficient (PCC) threshold that was used to convert co-expression data to networks and the coupling constant κ (kappa) that was used in OrthoClust analysis. We found that using a higher PCC threshold resulted in higher number of modules, which is expected because higher threshold in PCC resulted in fewer co-expression edges and smaller modules in the network. We found that using κ = 1 resulted in more modules as compared to using κ = 0. This is because that when κ = 0, the edges that represent homologous genes between two species are not used in the clustering analysis (see “Discussion” section). The parameter κ represents the relative weights of co-expression edges and homology edges in the module finding algorithm. We found that the number of modules does not increase dramatically when we set κ = 2 or 3 (see “Discussion” section for the effect of changing κ).

## Discussion

This protocol organized a number of computational tools into a pipeline to perform comparative transcriptome analyses. Depending on the species of interest, their available databases, or user preferences, there are multiple alternative bioinformatics tools for each step. For example, in searching for homologous genes, several other substitutable tools, such as OMA or OrthoFinder [10, 11], can be used instead of BLAST. A comprehensive comparison of these tools is out of the scope of this manuscript. Some databases or tools provide pre-computed homologous genes [8, 12]. Additional steps must be performed to ensure that the gene ids from OMA [10], OrthoFinder [11], or PLAZA [12] match the gene ids used in the expression analysis.

Moreover, in terms of clustering methods, we adapted OrthoClust, which provides a framework for module finding based on searching a lowest energy level of its cost function using simulated annealing. This approach can be expanded to other inputs such as by using many-to-many homologous relations in two species and by inferring modules across more than two species. There are other novel comparative transcriptome approaches that can be applicable to inter- or intra-species analysis (Table 4). For example, a modified K-mean clustering method was used for co-clustering transcriptome data from maize and rice after different segments of developing leaves [44]. In this work, a unified developmental model (UDM) was established by an iterative algorithm using approximately 3000 selected anchor genes which are homologous genes with similar expression patterns in two species. As compared to this method, our approach is more flexible because our approach does not require construction of UDM. Another example is the breadth-first search algorithm (TO-GCN) for time-ordered co-expression networks of transcription factors in maize [45]. In this work, maize and rice gene expressions used to construct co-expression networks separately, and maize specific TF-gene pairs were selected for experimental validation. In comparison to this work, we are using homologous information as edges in our network construction whereas TO-GCN focused on TF-target co-expression edges but did not include homologous relationships in their networks.

**Table 4:** Comparison of published methods that compare co-expression patterns across different plant species

Reference	Wang et al. [44]	Chang et al. [45]	This protocol
Species	Maize and rice	Maize	Arabidopsis and rice
Inputs	Transcriptomes from multiple segments of one tissue type in two species	Time series of transcriptomes from one tissue type under two conditions in one species	Time series of transcriptomes from one tissue type in two species
Methods	Correlate developmental gradients by constructing a unified developmental model (UDM) Co-cluster the fitted maize and rice gene expressions by using K-means clustering method	Construct co-expression networks by using the Pearson correlation coefficients (PCCs) Infer time-ordered gene co-expression networks (TO-GCNs) by the breadth-first search algorithm	Construct co-expression networks by using the Pearson correlation coefficients (PCCs) Integrate co-expression networks and pairs of orthologous genes, and infer network modules by using OrthoClust
Results	K clusters	TO-GCNs	Orthologous co-expressed modules
Tool availability	Data visualization is provided at: http://bar.utoronto.ca/Maize-Rice_eFP_Portal/	Pipeline software: https://github.com/petitmingchang/TO-GCN	Repository of this protocol: https://github.com/LiLabAtVT/CompareTranscriptome

Many genes in both species were not included in the RBH gene lists. This is because the criterion for identifying RBH genes is highly stringent. It requires that both genes in two species be the best BLAST hit in their respective species. This can be relaxed to identify k-best-hits in two species [6]. We have developed a script that can generate k-best-hits using BLAST results between any two species: OrthologousGenes_OneWayTopNBestHit.py, which is available in the github repository of this project.

The parameter κ is used to adjust the relative importance of the co-expression edges and homologous edges in network module finding algorithms (Fig. 5). When κ equals zero, the module finding method only finds co-expression modules and does not consider the effects of homologous edges. When κ is set to be higher than zero, homologous edges will be included in the module finding objective function. This can be verified by comparing the numbers of modules found when κ = 0 to numbers of modules found when κ > 0. The numbers of modules found when κ = 1 is two to three times the numbers of modules found when κ = 0. This result suggests that including homologous edges generates more modules across species, because, when κ = 0, all modules are from the same species. Comparing the numbers of modules from κ = 2 with κ = 1 and κ = 3 with κ = 2 suggest that increasing κ can further increase the number of modules. The PCC threshold also affects the number of modules identified (Fig. 5). For the same κ value, a higher PCC threshold always leads to more modules. This is expected as a co-expression network with higher PCC threshold contains fewer edges. Because of the reduced number of edges, the network is less connected and can be break into more modules as compared to the network generated with lower PCC threshold.

## Conclusions

In this article, we have presented a method to perform comparative transcriptome analysis. We provided a flexible workflow in publicly accessible scripts with detailed annotations. A users can use simple commands to execute the scripts following the instructions provided in the method section. From the sample analysis, we showed how orthologous relations in two species can be identified by reciprocal best hits (RBHs), what kinds of filtering methods can be applied to co-expression profiles, how to run the clustering methods, and how to visualize the results. Using this pipeline, we identified a module that includes genes that play important roles in embryo development in both species. We further explored this module by visualizing the inferred relationships of genes in the module as a network and by comparing expression patterns of the genes to understand conserved gene function between the two species. Using our proposed method, we were able to observe the conserved expression pattern and the example homologous genes in the example module from both species. In conclusion, our proposed method can be used to identify homologous genes with correlated expression patterns in two species.

## Methods

### Install software and download experimental data

All scripts used in this analysis can be obtained from github using the following command. (the git software is installed in most Linux systems by default. If git is not installed in your system, please refer to https://git-scm.com for installation instructions).

$ git clone https://github.com/LiLabAtVT/CompareTranscriptome.git ATH_GMA

All code blocks started with “$” are command line scripts that should be executed under a Linux terminal. All code blocks started with “>” are command line scripts that should be executed under an interactive R programming language console.

You can replace “ATH_GMA” with another folder name that better represents your project. All scripts in this project are tested under the project folder created by the “git clone” command (default ATH_GMA).

### Necessary resources

This protocol was tested under CentOS 7, which is a Linux-based operating system distribution. The steps described in this protocol can be used in most UNIX-like operating systems; this includes all major Linux distributions, and Mac OSX. For Windows users, the individual components of this protocol, such as BLAST, software used for RNA-Seq analysis, and programming languages R and Python, all have Windows-compatible executable files and can be used under Windows environments. In this protocol, we will install NCBI BLAST for the homology search step, STAR for read mapping and feature Counts for counting reads, and the R programming language and several packages for RNA-Seq and comparative transcriptome analysis.

### Set up a folder structure for data analysis

In this protocol, the reader can use the following commands to create the recommended folder structure (Fig. 2).

$ cd ATH_GMA

$ mkdir raw_data processed_data scripts results software

$ mkdir processed_data/bam processed_data/rc

Sequence and annotation files from databases should be downloaded to the *raw_data* folder. Software tools that will be used in this analysis can be saved and installed in the *software* folder. We recommend that the reader creates a folder named *bin* under the *software* folder such that the executable files can be copied to *software/bin* folder and add *software/bin* to the PATH environmental variable under the Linux environment. For experienced Linux users, software can also be installed in a user specified folder such as ~/bin or in a system wide folder. The reader can download scripts in github into the *scripts* folder. Intermediate output will be generated in the *processed_data* folder, and major input and output files for visualization will be saved in the *results* folder.

All scripts for this step are provided in Section2.1_setup_directory.sh in the scripts folder. The reader can set up the folder structure (Fig. 2) using the following command.

$ cd ATH_GMA

$ sh ./scripts/Section2.1_setup_directory.sh

### Software installation

We provide a script to download and install tools for RNA-seq analysis; readers can run the script in the project folder.

$ cd ATH_GMA

$ sh ./scripts/Section2.2_download_software.sh

A successfully installed tool will return version information when it is run with a -v or a --version option.

#### Install NCBI BLAST for identification of homologous genes

BLAST is a sequence similarity search tool [46]. The latest version of NCBI BLAST can be downloaded from the NCBI ftp site using the following link: ftp://ftp.ncbi.nlm.nih.gov/blast/executables/LATEST/. This folder contains precompiled executable files and installation files for Windows, Mac OSX, and Linux platforms. Because finding orthologous genes at a genome scale is computationally intensive, it is recommended to use a Linux workstation or computing cluster to perform the BLAST analysis.

For Linux users, the current pre-compiled executable is ncbi-blast-2.6.0 + -x64-linux.tar.gz.

For Mac users, the current installation file is ncbi-blast-2.6.0 + .dmg.

For Windows users, the current installation file is ncbi-blast-2.6.0 + -win64.exe.

A later version of BLAST should work as well with minor changes in the command line options. For Windows and Mac users, double click the downloaded file to install the program. For Linux users, one can use

$ tar –xvf ncbi-blast-2.6.0+-x64-linux.tar.gz

to extract the archive file. After extracting the files, move the executable files to a folder in the Linux search path.

#### Install tools for RNA-Seq data download

The following shows a sample script to download sra-tools and fastq-dump to download the raw sequencing data. The sequence read archive (SRA) database provides sra-toolkit, which is a suite of easy to use computational tools to download data from the database. To download the raw data from the SRA database, one needs to first install the sra-toolkit and use the fastq-dump utility program based on the SRA ids.

$ cd ATH_GMA/software

$ wget http://ftp-trace.ncbi.nlm.nih.gov/sra/sdk/current/sratoolkit.current-centos_linux64.tar.gz

$ tar -xzf sratoolkit.current-centos_linux64.tar.gz

$ ./sratoolkit.2.8.2-1-centos_linux64/bin/fastq-dump --version

#### Install tools for RNA-Seq data analysis

We will install the STAR [47] and featureCounts [48] software tools. STAR is a read mapper, and featureCounts can count the number of reads mapped to each gene in the genome. Both software tools were used here due to their speed and accuracy [49, 50]. Other alternative mappers can be used, and there are excellent review papers [50–52] that compare and summarize these different bioinformatics tools.

To download and install STAR and featureCounts, run the following scripts in the project folder.

$ cd Proj_CompTS_ATH_GMA/software

$ wget https://github.com/alexdobin/STAR/archive/2.5.2b.tar.gz

$ tar -xzf 2.5.2b.tar.gz

$ STAR-2.5.2b/bin/Linux_x86_64_static/STAR –version

$ wget https://sourceforge.net/projects/subread/files/subread-1.5.1/subread-1.5.1-Linux-x86_64.tar.gz/download

$ tar -zxvf download

$ subread-1.5.1-Linux-x86_64/bin/featureCounts -v

### Install R and DESeq2 packages for RNA-Seq data analysis

R is a programing language and environment for statistical data analysis [53]. We will use R to summarize RNA-Seq reads and to generate FPKM data. To install R, the reader should go to the Comprehensive R Archive Network (CRAN) (https://cran.r-project.org) to download the installer packages for their Windows, Mac OSX, or Linux system. For Linux users, R can be installed using the command line, and platform dependent package management systems. For example, to install R in CentOS 7 Linux, the user should simply type:

$ sudo yum install R

Scripts for installing R packages are provided in:

Section2.3_install_r_packages.R

This R script can be run on a Linux or MAC terminal by executing the script with the Rscript command directly, or a shell script that is a wrapper of the R script we provide.

$ cd ATH_GMA

$ Rscript ./scripts/Section2.3_install_r_packages.R

or

$ sh ./scripts/Section2.3_install_r_packages.sh

To install DESeq2 [54], the user should follow the instruction for the respective package. This package is part of the Bioconductor repository such that the installation should be performed using the Bioconductor installation script. The following commands are executed under the R environment and these commands are preceded by “>”. For commands that are executed under Linux terminals, these commands are preceded by “$”.

> source(‘https://bioconductor.org/biocLite.R’)

> biocLite(‘DESeq2’)

The installation script will detect the dependency of these two packages and install other required packages accordingly.

To install the OrthoClust package, the user should download the script for the OrthoClust package.

> setwd(“./software”)

> install.packages(“OrthoClust_1.0.tar.gz”, repos = NULL, type = “source”)

### Download protein and genome sequences for Arabidopsis and soybean

Sample scripts for download are provided in “Section2.4_download_data.sh”. All protein-coding sequences and genomic sequences for Arabidopsis can be downloaded from the Araport web site (www.araport.org). Araport is a data portal for Arabidopsis genomic research that hosts the latest genomic sequences and genome annotations for this model organism [55]. The web site requires free registration to access the download link to the protein sequences and genome annotation files. As of July 2017, the current version of the protein sequences file is “Araport11_genes.201606.pep.fasta.gz”. This name will likely be different for future versions of the protein sequences. We recommend that users download the latest version of the protein sequences, and record the actual download date and version of the sequence files for the purpose of reproducibility. The latest version of the genome sequence of Arabidopsis is “TAIR10_Chr.all.fasta.gz”. This file is unlikely to change because the genome assembly of Arabidopsis is likely to remain the same in the future. The latest version of the gene annotation file is “Araport11_GFF3_genes_transposons.201606.gtf.gz”.

All protein-coding sequences for soybeans can be downloaded from the DOE phytozome database (https://phytozome.jgi.doe.gov/pz/portal.html#!bulk?org=Org_Gmax). Phytozome is a data portal for plant and microbial genomes that hosts dozens of sequenced plant genomes and gene annotations [56]. This web site also requires free registration before data downloading. The latest version of soybean protein sequences is version 2.0 (downloaded in July 2017). The protein sequences and genomic sequences are “Gmax_275_Wm82.a2.v1.protein.fa.gz” and “Gmax_275_v2.0.fa.gz”. These names are likely to change with future versions of the genome and proteome annotation. The latest version of the gene annotation file is “Gmax_275_Wm82.a2.v1.gene_exons.gff3.gz”.

These files are in compressed fasta format and require decompression before use. Under the Linux command line, the following command can be used to decompress these *.gz files.

$ gunzip Araport11_genes.201606.pep.fasta.gz

$ gunzip Gmax_275_Wm82.a2.v1.protein.fa.gz

### Download raw data from published RNA-Seq experiments

Raw sequencing data can be downloaded from the NCBI Sequence Read Archive (SRA) (https://www.ncbi.nlm.nih.gov/sra). The embryo developmental data sets for Arabidopsis and soybean can be found in two bioprojects (PRJNA301162 for Arabidopsis [22] and PRJNA197379 for soybean [57]). For the Arabidopsis samples, RNA-Seq data were collected in triplicates at seven time points (7, 8, 10, 12, 13, 15, and 17 days after pollination). For the soybean samples, RNA-Seq data were collected in triplicates at ten time points (5, 10, 15, 20, 25, 30, 35, 40, 45, and 55 days, day 0 of the time course is 12 to 17 days after anthesis). Each sample is represented by a unique GSM id; for example, the three replicates of 7 days old Arabidopsis embryo samples are GSM1930276, GSM1930277, and GSM1930278. All 41 samples from this experiment are stored under a unique GSE id, GSE74692. Each sample is also represented by a unique SRA id. For example, the three replicates of 7 days old Arabidopsis embryo samples are SRR2927328, SRR2927329, and SRR2927330 from PRJNA301162.

$ fastq-dump --split-3 SRR2927328 --outdir ./raw_data

We suggest that the reader download the data into the raw data folder for further processing. To download large numbers of data sets, prepare a text file with all SRR ids for one species and run the following script in the project folder.

$ cd ATH_GMA

$ sh ./scripts/Section2.5_download_fastq.sh ./raw_data/PRJNA301162.txt ATH

$ sh ./scripts/Section2.5_download_fastq.sh ./raw_data/PRJNA197379.txt GMA

Depending on the size of sequencing data and network speed, this step may take a few hours. We provide a test file PRJNAtest.txt for the user to test the execution time for downloading one file. The time for downloading the entire data set can be estimated based on downloading this single file. We also provide the FPKM data for this particular data set so that the users do not need to download the original data to perform the analysis in this protocol. To perform the analysis using provided FPKM file, the user can start the analysis from a subsection of methods, *Identify orthologous co*-*expressed clusters using OrthoClust*.

### Identifying homologous genes between species

#### Identification of homologous pairs using BLAST

Analysis in this section can be performed using the following command:

$ cd ATH_GMA

$ sh ./scripts/Section3.2.1_BLAST.sh

*Step 1.* Merge the Arabidopsis protein fasta file and soybean protein fasta file using this Linux command:

$ cat Araport11.pep.fasta GLYMA2.pep.fasta>ATHGMA.pep.fasta

*Step 2.* Create the BLAST database:

$ makeblastdb -in ATHGMA.pep.fasta \

-out ATHGMA.blastdb \
-dbtype prot \
-logfile makeblastdb.log

The option –in specifies the input file name of the merged protein fasta file. The option –out specifies the BLAST database file name. The option –dbtype indicates the database is a protein database. The option –logfile is for recording error messages in case the process fails.

*Step 3.* Perform the BLAST search.

The Linux command used in this step is:

$ blastp -evalue 0.00001 \

-outfmt 6 -db ATHGMAX.blastdb \
-query ATHGMA.fasta>ATHGMA.pep.blastout

The option -evalue specifies the E value threshold. The option -outfmt is set to be 6, which is tab delimited format. The option -db is set to be the BLAST database built in step 3. The option -query uses the merged protein fasta files as input. The results of BLAST analysis are written in a file named ATHGMA.pep.blastout.

The output includes the following 12 tab-separated columns “*qseqid sseqid pident length mismatch gapopen qstart qend sstart send evalue bitscore”*. The meaning of these columns can be found using the BLAST help manual. The columns that will be used in downstream analysis are *qseqid* (query sequence id), *sseqid* (subject sequence id), and *evalue* (E value). We will filter BLAST results and only keep homologous genes with BLAST E value < 1e−5 [3, 26].

#### Obtaining reciprocal best hit (RBH) genes

We developed a Python script that can identify RBH genes from the above two species from BLAST results. The user can download this script from the github repository. To perform the analysis the user can use the following commands:

$ cd ATH_GMA

$ sh ./scripts/Section3.2.2_RBH.sh

### Gene expression data processing

Gene expression quantification includes three main steps: 1) read mapping; 2) read counting and 3) FPKM calculation. For this analysis, we follow a published protocol for expression processing [58].

*Step 1.* Create genome index by STAR

RNA-Seq reads have to be mapped to the respective reference genomes. To use STAR to map reads to the reference genome, the user needs to build a genome index using the following commands.

$ cd ATH_GMA

$ sh ./scripts/Section3.3.Step1.MakeIndex.sh

The following commands are used to create a genome index for Arabidopsis.

$ WORKDIR = $(pwd)

$ IDX = $WORKDIR/raw_data/ATH_STAR-2.5.2b_index

$ GNM = $WORKDIR/raw_data/TAIR10_Chr.all.fasta

$ GTF = $WORKDIR/raw_data/Araport11_GFF3_genes_transposons.201606.gtf

$ STAR --runMode genomeGenerate \

--genomeDir $IDX \
--genomeFastaFiles $GNM \
--sjdbGTFfile $GTF

The option --runMode indicates that the command is to create a genomic index. The option --genomeDir specifies the file name for the genome index. The option --genomeFastaFiles indicates the input fasta file for genomic sequences. The option --sjdbGTFfile is to provide a genome annotation file when creating the genomic index. A genome index will be created for each species.

*Step 2.* Read mapping by STAR

After creating genome indexes, the user needs to use STAR to map reads from each sample to the reference genome to generate a read mapping file using the following commands.

$ cd ATH_GMA

$ sh ./scripts/Section3.3.Step2.Mapping.ATH.sh

$ sh ./scripts/Section3.3.Step2.Mapping.GMA.sh

The Section3.3.Step2.Mapping.ATH.sh shell script is to map all Arabidopsis reads. The Section3.3.Step2.Mapping.GMA.sh shell script is to map all Soybean reads. In the SRA database, each sample has a unique SRR id. The following commands show one example of such SRR ids (SRR2927328). SRR2927328_1 and SRR2927328_2 represent two ends of paired reads.

$ STAR --genomeDir $IDX \

--readFilesIn $WORKDIR/raw_data/SRR2927328_1.fastq.gz $WORKDIR/raw_data/SRR2927328_2.fastq.gz \
--outFileNamePrefix $WORKDIR/processed_data/bam/SRR2927328/SRR2927328 \
--outSAMtype BAM SortedByCoordinate

The option --genomeDir specifies the file name for the genome index. The option --readFilesIn indicates the input fastq files for RNA-seq reads. Two files are provided for paired-end reads. The option --outFileNamePrefix is to provide the directory for output data. The option --outSAMtype BAM indicate the output file should be a BAM file. SortedByCoordinate sets the output data to be sorted by the order of where the read is mapped to the chromosome.

*Step 3.* Read counting with featureCounts

To count reads with featureCounts, the user can use the following command:

$ cd ATH_GMA

$ sh ./scripts/Section3.3.Step3.ReadCount.ATH.sh

$ sh ./scripts/Section3.3.Step3.ReadCount.GMA.sh

For this step, featureCounts will calculate how many reads map to each gene region. For simplicity, we only count uniquely mapped reads and only summarize read counts at the gene level. Other software can be used to summarize expression at isoforms levels. The following commands are for counting reads for a single file.

$ WORKDIR = $(pwd)

$ GTF = $WORKDIR/raw_data/Araport11_GFF3_genes_transposons.201606.gtf

$ BAM = $WORKDIR/processed_data/bam

$ RC = $WORKDIR/processed_data/rc

$ featureCounts -t exon \

-g gene_id \
-p \
-a $GTF \
-o $RC/SRR2927328.readcount.txt \
- $BAM/SRR2927328/SRR2927328Aligned.sortedByCoord.out.bam

The option -t exon indicates that only reads mapped to exons are counted. The option -p indicates the input reads are paired-end reads. The option -a provides the location of the genome annotation file. The option -o specifies the output file location. The last parameter is the file name of the read mapping file (BAM file).

*Step 4.* FPKM calculation using DESeq2

For this step, R scripts will be used to summarize gene expression level in fragments per kilobase pairs per million reads (FPKM). To calculate FPKM using DESeq2 package, we performed the following four steps: (1) merging read counts from different files into one single file; (2) differential expression analysis using DESeq2; (3) FPKM calculation; and (4) average FPKM calculation across replicates. These steps can be performed using a unified R script: Section3.3.Step4.FPKM.R. To run this script, the user needs to provide a table that summarizes the replicate structure of the samples. Example tables (PRJNA301162.csv for Arabidopsis and PRJNA197379.csv for soybean) are provided in the processed_data folder.

To run the unified R script for FPKM calculation, use the following commands:

$ cd ATH_GMA

$ Rscript ./scripts/Section3.3.Step4.FPKM.R ./processed_data/fpkm/GMA

$ Rscript ./scripts/Section3.3.Step4.FPKM.R ./processed_data/fpkm/ATH

This script requires multiple input files to be present in the working directory. These files include a file that describes the design matrix of the experiment and the read count files generated in Step 3. More descriptions of the input file formats are included in the annotation of the R script.

*Step 5.* Co-expression networks from gene expression profiles

Expression data will be summarized and converted to gene co-expression networks. The input data include data matrices with averaged and normalized FPKM values. In this protocol, we use genes in metabolic pathways of seed development [28, 42]. Other methods can be used to filter genes before the analysis, for example, only keep genes with high variations across conditions (variance > 0.5) or genes with minimum gene expression level (FPKM ≥ 0.5 from any conditions). Finally, gene co-expression matrices were calculated using the Pearson Correlation Coefficient (PCC) of the FPKM values between the filtered sample genes for each species. The gene co-expression matrices were converted into co-expression networks with an edge list by treating each gene as a node and a PCC values as an edge between genes after the cut-off with p value < 0.001 and Pearson correlation coefficient > 0.99. To generate co-expression networks from gene expression profiles, the following commands were used.

$ cd ATH_GMA

$ Rscript ./scripts/Section3.3.Step5_FPKM2NETWORK.R

### Identify orthologous co-expressed clusters using OrthoClust

#### Overview of the OrthoClust method

Simple approaches can be used to identify conserved co-expression genes across different species. For example, one can first cluster gene expression in two species separately, and, for each pair of cluster combinations, one can find whether the pairs of clusters share significantly large numbers of homologous genes using appropriate statistical tests such as Fisher’s exact test. OrthoClust [29] is a global approach in which the process of co-expression clustering finding and homology detection is integrated into the same objective function. The objective function *H* is defined as

$$H = - \left( {\mathop \sum \limits_{{i,j \in S_{1} }} \varLambda_{ij}^{1} \delta_{{\sigma_{i} \sigma_{j} }} + \mathop \sum \limits_{{i,j \in S_{2} }} \varLambda_{ij}^{2} \delta_{{\sigma_{i} \sigma_{j} }} + \kappa \mathop \sum \limits_{{\left( {i,j^{\prime}} \right) \in {\rm O}\left( {S_{1} ,S_{2} } \right)}} w_{{ij^{'} }} \delta_{{\sigma_{i} \sigma_{{j^{\prime}}} }} } \right)$$

where *S*_*N*_ is the sets of genes for a species and a subscript of *S* (N = 1 or 2) corresponds to the species respectively. The variables *i* and *j* are individual genes of a species or nodes on a network. $\varLambda_{ij}^{N}$ denotes a modularity score from gene *i* and *j*, that is a difference between the real number of edges and the expected number of edges. $\delta_{{\sigma_{i} \sigma_{j} }}$ is for a module label. If genes *i* and *j* have the same module label, $\delta_{{\sigma_{i} \sigma_{j} }}$ = 1, and, if not, $\delta_{{\sigma_{i} \sigma_{j} }}$ = 0. A coupling constant, $\kappa$ (kappa) controls overall impact of orthology relations on the objective function, and a weight, $w_{{ij^{'} }}$ is for orthology relations coming from the number of orthologous genes between two species. The objective function *H* will return lower values when orthologous genes are assigned into the same module.

This approach translates orthologous co-expression finding into a network module finding problem. The objective function includes three components: two components represent the goodness of the expression clustering results and one component represents the effect of homologous genes across species. The parameter κ can be adjusted to increase or decrease the contribution of homologous genes in the clustering processes.

#### Steps for OrthoClust analysis

To perform OthoClust analysis, we require three input data files (Table 2): (1) the gene co-expression network from soybean; (2) the gene co-expression network from Arabidopsis; and (3) the orthologous gene pairs between two species.

These files require a specific format for the OrthoClust engine to analyze. The user can use the following R command to perform the clustering analysis:

> library(OrthoClust)

> OrthoClust2(Eg1 = GMX_edgelist, Eg2 = ATH_edgelist, \

- list_orthologs = GA_orthologs, kappa = 3)

We provide a wrapper script that will read three input files: a list of edges from Arabidopsis, a list of edges from soybean, and a list of RBH gene pairs from two species. To perform OrthoClust analysis, the user can simple use the following commands:

$ cd ATH_GMA

$ Rscript ./scripts/Section3.4.Step1_OrthoClust.R

This script will generate three files. Orthoclust_Results.csv contains information regarding modules assignment for each gene. Orthoclust_Results_Summary.csv includes number of genes assigned to each module. Orthoclust_Results.RData contains multiple R objects that will be used in the visualization step.

#### Visualization of OrthoClust results as a network

To generate these files for Cytoscape visualization, the user can use the following command.

$ cd ATH_GMA

$ Rscript ./scripts/Section3.4.Step2_CytoscapeInput.R

*Step 1.* To Import three files on Network Browser, we can first start from the Cytoscape menu bar and follow these choices: File > Import > Network > File. After you select one of three input files, the popup window with “Import Network From Table” title appears. You can see two columns with gene names in the middle of the window. Next, to change attributes of columns, click the first line of each column and choose either Source Node or Target Node from the menu. Since three edge lists do not have direction, the two columns from each input file can be assigned into either source or target nodes. After that, we change an option for column names from Advanced Options at the bottom left of the window. On the new popup window, we can uncheck “Use first line as column names”, since we do not have headers in the input files. Finally, you can see two column names, Column1 and Column2 with different icons of attributes, and the remaining parts of the preview are gene names. You can repeat these steps for each of the input files.

*Step 2.* With three imported networks, we can integrate data sets of co-expression networks with homologous relations using the Union function. To do that, select three networks on the network tab on the control panel (click one network and click the other two networks while pressing Command), and move to Cytoscape’s menu bar: Tools > Merge > Networks.

In the popup window for Advanced Network Merge, we should choose the Union button, select three networks from Available Networks, and then click the right-facing arrow acting for Add Selected. After that you can find that three networks are now on Networks to Merge, and you can click the Merge button to merge three networks.

The name of the merged network will appear with the total number of merged nodes and edges on the Network tab on the control, and usually it is automatically visualized on the Cytoscape canvas.

*Step 3.* To express properties of networks (species information, source of edges such as co-expression networks or homologous relations), we can customize visual attributes of the merged network. To do that, on the Select tab on the control panel, we can click the + icon below Default filter and choose Column Filter to add the new condition. From the Choose column drop-down list, you can select Node: name or Edge: name and type a prefix of each species (“AT” for Arabidopsis genes, or “Glyma” for soybean genes). This filter applies to visualization of the merged automatically, so you can see highlighted nodes on the Cytoscape canvas.

There are several ways to change visualization properties of the selected components. First, we can set Bypass Style for the selected nodes or edges such as Fill Color and Size for properties of nodes, or Stroke Color and Line Type for properties of edges. To do this, move your mouse pointer on one of the highlighted nodes, right-click, and then select Edit > Bypass Style > Set Bypass to Selected Nodes on the popup menu. The control panel on the left side will be automatically changed to the Style tab, and you can see three subtabs: Node, Edge, and Network on the bottom of the interface. Second, we can apply different Layouts with these selected nodes or all nodes from Cytoscape menu bar Layout.

#### Visualization of OrthoClust results as expression profiles

We also provide a R scripts to directly visualize gene expression patterns for orthologous co-expression modules into a figure. This script will generate gene expression profile plots of genes in a selected module including homologous genes of these genes along different time points. It can also highlight expression pattern of interesting genes with different color line. The figure can be generated by the following commands:

$ cd ATH_GMA

$ Rscript ./scripts/Section3.4.Step2_CytoscapeInput.R

## Data Availability

The data sets and software supporting the conclusions of this article are available in the Github repository (https://github.com/LiLabAtVT/CompareTranscriptome).
